# High fat diet and PCSK9 knockout modulates lipid profile of the liver and changes the expression of lipid homeostasis related genes

**DOI:** 10.1186/s12986-023-00738-z

**Published:** 2023-03-31

**Authors:** Krisztina Németh, Blanka Tóth, Farkas Sarnyai, Anna Koncz, Dorina Lenzinger, Éva Kereszturi, Tamás Visnovitz, Brachyahu Meir Kestecher, Xabier Osteikoetxea, Miklós Csala, Edit I. Buzás, Viola Tamási

**Affiliations:** 1 Department of Genetics, Cell- and Immunobiology, Semmelweis University, Nagyvárad Tér 4, Budapest, 1085 Hungary; 2 ELKH-SE Translational Extracellular Vesicle Research Group, Nagyvárad Tér 4, Budapest, 1085 Hungary; 3 Department of Molecular Biology, Semmelweis University, Tűzoltó U. 37-47, Budapest, 1094 Hungary; 4 Department of Inorganic and Analytical Chemistry, Budapest University of Technology and Economics, Műegyetem Rkp. 3, Budapest, 1111 Hungary; 5 Department of Plant Physiology and Molecular Plant Biology, Eötvös Loránd University, Pázmány Péter Sétány 1/A, Budapest, 1117 Hungary; 6 HCEMM-SE Extracellular Vesicle Research Group, Nagyvárad Tér 4, Budapest, 1085 Hungary

**Keywords:** Liver, Dyslipidemia, Hepatosteatosis, PCSK9, LDLR, CD36, ANXA2

## Abstract

**Background:**

High fat diet (HFD) increases the likelihood of dyslipidemia, which can be a serious risk factor for atherosclerosis, diabetes or hepatosteatosis. Although changes in different blood lipid levels were broadly investigated, such alterations in the liver tissue have not been studied before. The aim of the current study was to investigate the effect of HFD on hepatic triglyceride (TG), diglyceride (DG) and ceramide (CER) levels and on the expression of four key genes involved in lipid homeostasis (*Pcsk9, Ldlr, Cd36* and *Anxa2*) in the liver. In addition, the potential role of PCSK9 in the observed changes was further investigated by using PCSK9 deficient mice.

**Methods:**

We used two in vivo models: mice kept on HFD for 20 weeks and PCSK9^−/−^ mice. The amount of the major TGs, DGs and CERs was measured by using HPLC–MS/MS analysis. The expression profiles of four lipid related genes, namely *Pcsk9, Ldlr, Cd36* and *Anxa2* were assessed. Co-localization studies were performed by confocal microscopy.

**Results:**

In HFD mice, hepatic PCSK9 expression was decreased and ANXA2 expression was increased both on mRNA and protein levels, and the amount of LDLR and CD36 receptor proteins was increased. While LDLR protein level was also elevated in the livers of PCSK9^−/−^ mice, there was no significant change in the expression of ANXA2 and CD36 in these animals. HFD induced a significant elevation in the hepatic levels of all measured TG and DG but not of CER types, and increased the proportion of monounsaturated vs. saturated TGs and DGs. Similar changes were detected in the hepatic lipid profiles of HFD and PCSK9^−/−^ mice. Co-localization of PCSK9 with LDLR, CD36 and ANXA2 was verified in HepG2 cells.

**Conclusions:**

Our results show that obesogenic HFD downregulates PCSK9 expression in the liver and causes alterations in the hepatic lipid accumulation, which resemble those observed in PCSK9 deficiency. These findings suggest that PCSK9-mediated modulation of LDLR and CD36 expression might contribute to the HFD-induced changes in lipid homeostasis.

**Supplementary Information:**

The online version contains supplementary material available at 10.1186/s12986-023-00738-z.

## Background

Dyslipidemia is a complex medical condition that is characterized by increased concentration of low-density lipoprotein cholesterol (LDL-C) and triglyceride (TG) and/or low level of high-density lipoprotein (HDL) in the blood. The above-mentioned dysfunction is considered to account for a high risk of metabolic syndrome, obesity, non-alcoholic fatty liver disease (NAFLD) or combined hyperlipidemia [1, 2]. According to the WHO report (2008), Europe is leading with the prevalence of a total cholesterol level ≥ 4.9 mmol/L of 54% and is followed by North and South America (48%) [3]. Moreover, based on 2019 WHO statistic, dyslipidemias became one of the most important risk factors for two global causes of death: stroke and ischemic heart disease [4].

Cholesterol (C) and TG levels in the plasma are highly associated with LDL particle number. LDL binds to several receptors on the surface of hepatocytes, such as low-density lipoprotein receptor-related protein 1 (LRP1 or CD91), fatty acid translocase scavenger receptor (CD36) and low-density lipoprotein receptor (LDLR), the latter being the most important. There are two types of LDLR, soluble and membrane-bound [5]. Studies show that soluble LDLR levels correlate with several lipoprotein parameters [6] and that hepatic LDLR levels inversely correlate with plasma LDL-C concentration [7]. Hepatic LDLR activity is modulated by different hormones and growth factors, such as insulin [8] and estradiol [9]. The key regulator of LDLR gene expression at the transcriptional level in hepatocytes is sterol regulatory element-binding protein 2 (SREBP2) [10].

Degradation and recirculation of LDLR to the plasma membrane surface is highly affected by the proprotein convertase subtilisin/kexin type 9 serine protease (PCSK9). PCSK9 binds the EGF-A domain of LDLR through its catalytic domain [11], and thus directs LDLR to the endocytic pathway towards the lysosomes, which prevents the recycling of the receptor protein. The significance of this PCSK9-LDLR interaction in the modulation of blood cholesterol level was emphasized by genetic studies. In humans, PCSK9 loss-of-function mutations are associated with hypocholesterolemia while gain-of-function mutations are associated with a familial hypercholesterolemia [12, 13]. As a regulator of LDLR, PCSK9 has been recognized as a promising therapeutic target in dyslipidemia related diseases, and various therapeutic strategies [14] have been studied for patients where statins have no/insufficient pharmacological effect [15].

In addition to LDLR, PCSK9 also enhances the degradation of CD36 [16]. CD36 has many lipid related functions in the liver, such as binding free long-chain fatty acids or LDL and facilitating their transport into the cells [17]. Similarly to LDLR, the level of CD36 is increased in hepatocytes under lipotoxic conditions or in NAFLD [18].

The PCSK9-mediated control of LDLR recycling is also influenced by another protein, Annexin A2 (ANXA2). Allosteric regulation by ANXA2 causes a conformational change in PCSK9 protein that hampers its binding to LDLR [19]. Recent studies showed that LDLR levels were decreased by 20% in the liver of ANXA2^−/−^ mice and the reduction was even more marked in tissues like adrenal glands and colon, which are known to be rich in ANXA2 and resistant to PCSK9 effect [20, 21].

Lipids accumulate in the liver mainly as TGs [22]. Under dyslipidemic conditions, the type and quantity of accumulated lipids change in hepatocytes, which influence the outcome and the severity of liver diseases [23]. For instance, ceramides (CERs) modulate insulin sensitivity, cause metabolic derangement and in high concentration, stimulate cell death [24–26]. Additionally, it has been reported that the lipid composition of the diet modulated the lipid content and oxidation of LDL [27] and various diets such as HFD or an olive oil rich diet can change the lipid profile in hepatocytes differently. Furthermore, some lipids are more toxic than others and can induce oxidative and inflammatory processes [27]. Thus, a shift toward toxic lipids can promote the progression and severity of dyslipidemia-associated diseases, such as NAFLD.

Moreover, hepatic uptake of lipids largely influences the composition and amount of plasma lipids. Inhibition of PCSK9 significantly decreased plasma levels of several lipid classes, including sphingolipids (dihydroceramides, glucosylceramides, sphingomyelines, ceramides), cholesteryl esters and free cholesterol, while inflammation related eicosanoids, were not altered. Recent studies show that many lipid homeostasis-related genes are modulated in mice fed with HFD. For example, an alteration in the expression and activity of serine palmitoyltransferase entails changes in net accumulation of dihydrosphingosine and dihydroceramide [28].

The aim of the present study was to investigate how chronic exposure to an obesogenic HFD changes the lipid content and expression of certain lipid homeostasis related genes in the liver. Since the different lipids do not weigh equally in the development of liver toxicity, we considered it important to measure the amount of selected lipids (subtypes of TGs, DGs and CERs) by mass spectrometry in liver tissue of mice on HFD.

It is known that PCSK9 has a role in the regulation of lipid accumulation in the liver [29], but its effects have been poorly characterized. Therefore, we also aimed to investigate the alterations in the lipid profile of PCSK9^−/−^ mice. While lipid accumulation in hepatocytes is affected by many proteins, we chose to assess changes in two major lipid uptake receptors (LDLR, CD36) as the recycling of these proteins is controlled by PCSK9 and its natural inhibitor ANXA2. Our findings suggest that the HFD-induced changes in lipid homeostasis can be partly attributed to the PCSK9-mediated modulation of LDLR and CD36 expression. Investigation of LDLR/CD36-PCSK9-ANXA2 axis may contribute to the understanding of liver diseases and open new perspectives for the development of novel therapeutic strategies.

## Methods

### Mice

Mouse experiments followed the Council Directive of the European Union (86/609/EEC), and ethics approval was obtained from the Semmelweis University’s Institutional Animal Care and Use Committee (PE/EA/1655-7/2018). Male, C57BL/6 and PCSK9^−/−^ (C57BL/6 background, n = 5 in each group) mice were purchased from the Jackson Laboratory. Mice were maintained at the animal facility of the Department of Genetics, Cell- and Immunobiology, with a normal light cycle (12–12 h) and fed ad libitum. At 6 weeks of age, wild-type (WT) C57BL/6 mice were randomly allocated into two groups and fed either a HFD (45 kcal% fat, D12451, Research Diets, USA) or a normal mouse diet for 20 weeks. Mice kept on control diet or HFD, as well as 12-week-old WT and PCSK9^−/−^ mice were sacrificed with CO_2_, 5 h after food deprivation. Liver pieces were collected and were snap frozen in liquid nitrogen and stored at − 80 °C until further use.

### Evaluation of hepatic lipid accumulation by HPLC–MS/MS

Two pieces of 1–2 mg weight were collected from each liver of five mice, and their CER, DG and TG contents were measured and normalized to proteins according to Sarnyai et al. [30].

Briefly, a piece of liver was lysed in buffer containing 0.1% SDS, 5 mM EDTA, 150 mM NaCl, 50 mM Tris, 1% Tween 20, 1 mM Na_3_VO_4_, 1 mM PMSF, 10 mM benzamidine, 20 mM NaF, 1 mM pNPP, and a protease inhibitor cocktail. Lysates were centrifuged (10 min, 10,000 rpm, 4 °C). The protein concentration of the supernatant was determined by Pierce BCA Protein Kit Assay (ThermoFisher, USA). An additional excel file shows the protein concentration of the liver samples (Additional file 1).

Liver samples were suspended in a mixture of methanol–isopropanol (1:1 ratio) containing ceramide 17:0 (500 ng/mL), DG 17:0/17:0 d5 (1 µg/mL) and TG 17:0/17:0/17:0 (1 µg/mL) internal standards. The samples were homogenized with an ultrasonic sonotrode and centrifuged (10 min, 13,400 rpm, 24 °C). The supernatants were transferred to vials for HPLC–MS/MS analysis.

5 µL samples were injected in the HPLC (Agilent 1100). A Kinetex^®^ 5 µm, C8 100 Å, LC (100 × 3 mm) column was used with a gradient elution of 10 mM ammonium-acetate (mobile phase A) and methanol (mobile phase B) and isopropanol (mobile phase C): 0 min at 10% A, 90% B, 0% C; 5 to 7 min at 5% A, 95% B, 0% C; 14 to 16 min at 5% A, 55% B and 40% C; 17 to 23 min at 10% A, 90% B, 0% C. 1. CER, DG and TG species were detected using a triple quadrupole mass spectrometer (SCIEX 3500). The instrument was used in positive multiple reaction monitoring mode. The ion spray temperature was set to 450 °C and the voltage to 5500 V. Quantitative analysis was based on internal standard method by using non-physiological metabolite analogues, i.e., ceramide 17:0 (500 ng/mL), DG 17:0/17:0 d5 (1 µg/mL) and TG 17:0/17:0/17:0 (1 µg/ml) internal standards. An Additional file 1 shows the measured lipid concentration of the liver samples (Additional file 1).

### Gene expression analysis

For gene expression analysis, 40–50 mg liver sample was homogenized with a micropestle and syringe in Qiazol Lysis Reagent (QIAGEN, Germany). Total RNA was purified using a Blood/Cell Total RNA Mini Kit (Geneaid, Taiwan). The quantity of the RNA was measured using a Nanodrop-1000 (ThermoFisher, USA). We used SensiFAST cDNA Synthesis Kit (Bioline, UK) to prepare cDNA from 1 µg of total RNA. Quantitative real-time PCR (RT-qPCR) was performed on the 7900 HT Fast Real-Time PCR System (Applied Biosystems, USA), using SensiFAST Probe Hi-ROX Kit (Bioline, UK) according to the instructions of the manufacturer. Briefly, each reaction was performed in the final volume of 10 µL, including 50 ng of cDNA (in 4.5 µL), 0.5 µL of Taqman assay and 5 µL of SensiFAST Probe Hi-ROX mix. Taqman assays were used for *Gapdh* (Mm9999915_g1), *Cd36* (Mm00432403_m1), *Ldlr* (Mm01177349_m1), *Anxa2* (Mm00500307_m1) and *Pcsk9* (Mm01263609_g1). The fold changes of mRNA were calculated using 2^−*ΔΔ*Ct^ method, normalized to *Gapdh* RNA internal control.

### Western blot analysis

Liver LDLR, CD36 and ANXA2 levels were assessed by Western blot. Liver lysate was prepared from 15 to 25 mg of tissue. Tissue pieces were homogenized with a micropestle rod and syringe in lysis buffer (Cell Lysis Buffer, Cell Signaling, USA) supplemented with protease inhibitors (Complete Protease Inhibitor, EDTA-free, Sigma, USA). Samples were incubated on ice for 30 min and regularly vortexed at maximum speed (30 rpm). The lysates were sonicated for 10 min in a cooled sonicator and the remaining tissue pieces were removed by pelleting at 14,500 g for 10 min at 4 °C. The supernatant was aspirated to avoid floating fat and the protein content of the samples was determined by Micro BCA assay (ThermoFisher, USA).

Proteins were separated on a 10% polyacrylamide gel (acrylamide/bisacrylamide ratio 37.5:1) by size using a MiniProtean (BioRad, USA) gel running system. For easier solubilization of membrane proteins, protein samples were mixed with equal volumes of 0.1% TritonX^®^-100 and Leammli buffer (3x). Thirty µg of protein/sample was loaded into the gel pockets. Following electrophoretic separation, the proteins were applied to a polyvinylidene fluoride membrane (Serva, Germany). The membrane was blocked with 5% low fat milk powder for 1 h at room temperature with rotation. Primary antibodies were incubated overnight with the membrane at 4 °C by rotation in 5% low fat milk powder. The following antibodies were used: monoclonal rabbit anti-Annexin A2 (1:1,000, clone: JA42-30, ThermoFisher, USA), monoclonal mouse anti-CD36 (1:500, clone: D-2712, ThermoFisher, USA), rabbit monoclonal anti-LDLR (1:1,000, clone: SJ0197, ThermoFisher, USA), polyclonal rabbit anti-actin (1:2,000, Sigma, USA). After the removal of primary antibody in excess, membranes were incubated with HRP-labeled polyclonal anti-rabbit IgG and anti-mouse IgG secondary antibodies (Abcam, UK, 1:20,000) for 40 min at room temperature in 1% low-fat milk powder. A solution of ECL Western Blotting Substrate (ThermoFisher, USA) was used as HRP substrate. The chemiluminescent signal was detected using an Imager CHEMI Premium (VWR, USA) image analysis system. The optical densities of the obtained bands were determined with ImageJ software for 5 samples. Original blots and two additional samples are presented as Additional file 2: Figs. S2 and S3. Each value was normalized to the actin internal control protein. All antibodies used in these experiments are listed in Additional file 2: Table S1.

### Evaluation of hepatic PCSK9 concentration

Liver PCSK9 protein levels were determined by ELISA. Protein lysate was prepared as recommended by the LEGEND MAX™ Mouse PCSK9 ELISA Kit (BioLegend, USA). Liver tissue (30–40 mg) was frozen in liquid nitrogen and was homogenized in ice cold PBS supplemented with a protease inhibitor (Complete Protease Inhibitor with EDTA, Sigma, USA) using a micropestle rod and syringe. The remaining larger pieces of tissue were removed by centrifugation (10,000 g, 15 min, 4 °C). The protein content of the supernatant was determined with a Micro BCA assay (ThermoFisher, USA).

### Cell culture

HepG2 (ECACC 85,011,430) human hepatocellular carcinoma cells were purchased from Merck (Germany) and were cultured in DMEM medium (low glucose, 1 g/L), supplemented with 10% fetal bovine serum (FBS, Gibco, USA) 1,000 U/L penicillin, 1,000 µg/L streptomycin, 2 mmol/L L-glutamine (Sigma). Cells were maintained at 37 °C in a humidified atmosphere containing 5% CO_2_. The cells were plated either in 24-well TC-treated plate (Eppendorf, Germany) or on gelatin-fibronectin coated coverslipes (12 mm, VWR, USA) with a density of 5 × 10^4^ cells/well. After the seeding, we allowed the cells to reach 55–60% confluency (~ 3 days) (Additional file 2: Fig. S4).

FBS is known to decrease the expression of proteins involved in lipid metabolism [31]. For this reason, the FBS content of the medium was reduced to 2% before any further analysis. At 16 and 40 h after the medium change, the confluence of the cells was determined with ImageJ software. The cells were then detached from the surface of the plate and the cell number and cell viability were determined by hemocytometer with Trypan blue staining.

### Assessment of the PCSK9 secretion by HepG2 cells

PCSK9 production of HepG2 cells was quantified 16 and 40 h after we decreased the FBS content of the medium. PCSK9 secretion was measured from the conditioned medium using a LEGEND MAX™ Human PCSK9 ELISA Kit (BioLegend, USA).

### Co-localization of PCSK9 with LDLR, CD36 and ANXA2

For immunocytochemistry, HepG2 cells were plated on fibronectin-gelatin coated coverslips (12 mm, VWR, USA). Cells were fixed with 4% PFA for 40 h after we reduced FBS content of the medium. The plasma membrane was stained with lactadherin (Haematologic Technologies, labelled with Atto-488 Fast Conjugation Kit, Abcam) as described previously [32]. After a postfixation step with 4% PFA, the samples were blocked with PBS containing 10% FBS, supplemented with 0.3% Triton X-100 (Molar Chemicals Kft., Hungary). Primary antibodies were incubated overnight at 4 °C in 1:100 dilution (monoclonal mouse anti-CD36, clone: FA6-152, Abcam, UK; monoclonal rabbit anti-LDLR, clone: SJ0197, ThermoFisher; monoclonal rabbit anti-Annexin A2, clone: JA42-30, ThermoFisher; polyclonal goat anti-PCSK9, Sigma; monoclonal mouse anti-PCSK9, clone: 2F1, ThermoFisher). After washing out the primary antibodies, secondary antibodies were incubated for 1 h in 1:1000 dilution at room temperature with shaking (goat polyclonal anti-rabbit IgG-AF700, ThermoFisher; goat polyclonal anti-mouse IgG-eF570, ThermoFisher; rabbit polyclonal anti-goat IgG-Cy3, Sigma, USA; goat polyclonal anti-mouse Cy5, ThermoFisher). Samples were washed with PBS, distilled water and covered with ProLong™ Gold antifade reagent with DAPI (ThermoFisher). The slides were examined with Leica TCS SP8 Confocal Laser Scanning microscope (Leica, Germany). All antibodies used in these experiments are listed in Additional file 2: Table S1. Co-localization rate of PCSK9 protein with LDLR, CD36 and ANXA2 was analyzed in Leica Application Suite X (LAS X) software.

### Statistics

Statistical analyses were performed using Prism 7.00 (GraphPad Software Inc. USA). Results are expressed as mean ± standard deviation (SD). Normal distribution of data was evaluated with Shapiro–Wilk normality test. Differences between normally distributed groups were evaluated by unpaired T-test, multiple T-test or one-way ANOVA combined with Tukey’s multiple comparisons test. A probability value of *p* < 0.05 was considered statistically significant.

## Results

### Hepatic lipid profile of WT mice on control diet and HFD

**Fig. 1:**
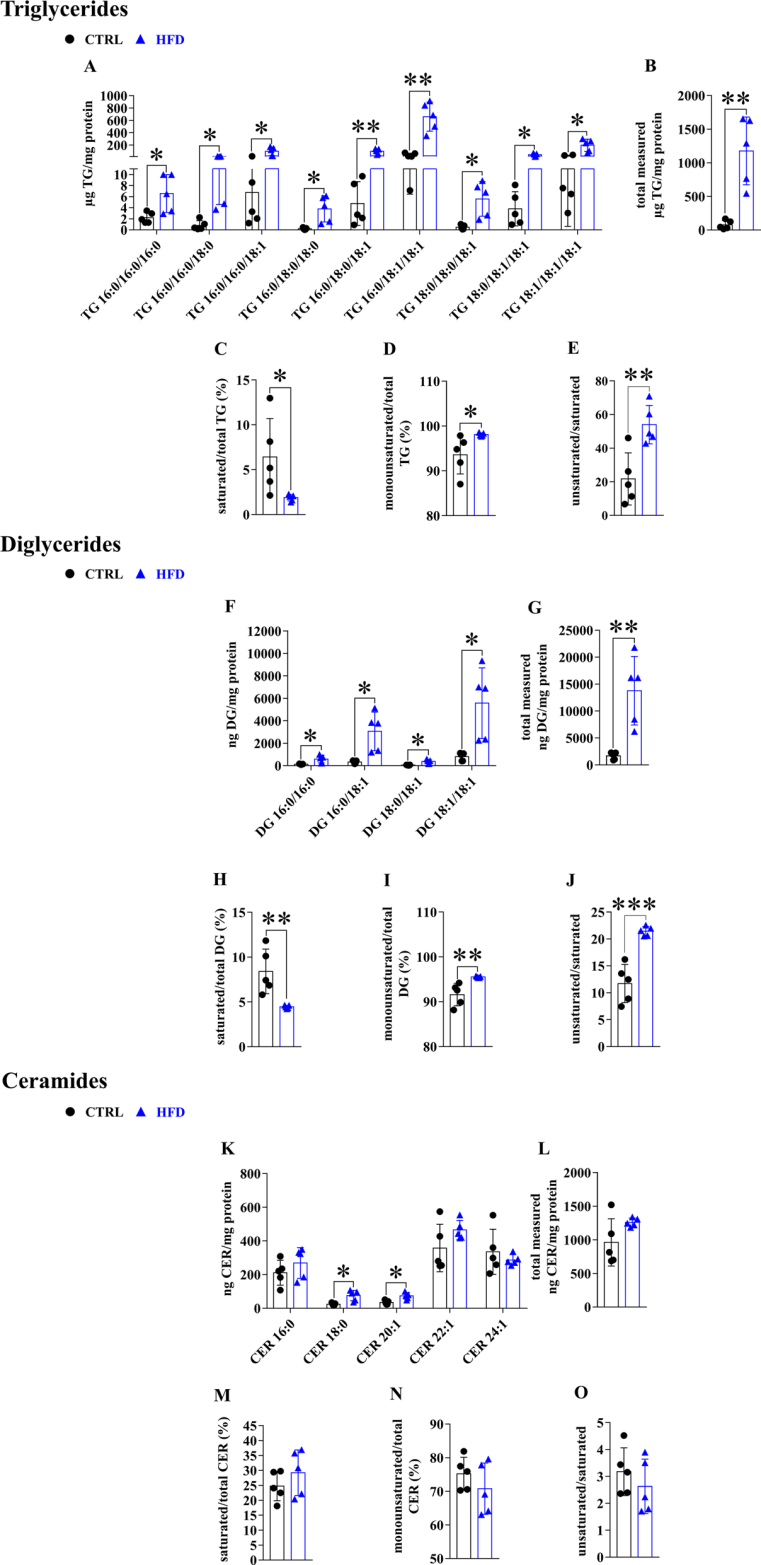
Lipid accumulation in the liver after 20 weeks on HFD. Lipid composition of the liver was analyzed by HPLC–MS/MS after 20 weeks on control or HFD diet. The concentration of TG, DG and CER subtypes (**A, F, K**), their total amount (**B, G, L**), the proportion of saturated and monounsaturated TGs, DGs and CERs relative to total amount (**C, D, H, I, M, N**) and the ratio of monounsaturated and saturated lipids were determined (**E, J, O**). Values are reported as mean ± SD; n = 5 in each group. The *p* value was determined by multiple T-test (**A, F, K**) or unpaired T-test (all the others). *p < 0.05, **p < 0.01, ***p < 0.001

HFD is known to increase lipid accumulation in the liver. WT mice were kept on HFD for 20 weeks before the lipid composition of their livers was analyzed by HPLC–MS/MS. Among the lipid types detected, 1,2-dioleyl-3-palmitoylglycerol (52:2), 1,2,3-trioleylglycerol (54:3), 1,2-dioleylglycerol (36:2), 1-stearyl-2-oleylglycerol (34:1), as well as docosenoic acid (22:1) and tetracosenoic acid (24:1) containing CERs predominated in both the control and HFD groups (Fig. 1).

After 20 weeks on HFD diet, there was a significant increase in the detected TG and DG subtypes and in their total quantity (Fig. 1A, B, F and G). The levels of CERs containing stearic acid (18: 0) and eiocosenoic acid (20:1) were significantly increased (Fig. 1K), but the total concentration of CERs did not change (Fig. 1L). We examined the relative contribution of the saturated and monounsaturated fatty acids to the total lipid content of the liver tissue. The proportion of saturated TGs and DGs decreased significantly (Fig. 1C, H), while the proportion of monounsaturated TGs and DGs increased (Fig. 1D, I). No such trend was observed when the CERs were analyzed (Fig. 1M, N). The ratio of monounsaturated/saturated fatty acids was significantly higher in TGs and DGs in the HFD group (Fig. 1E, J), but no change was observed in case of CERs (Fig. 1O).

### Expression of PCSK9, LDLR, CD36 and ANXA2 in the liver of WT mice fed with HFD

**Fig. 2:**
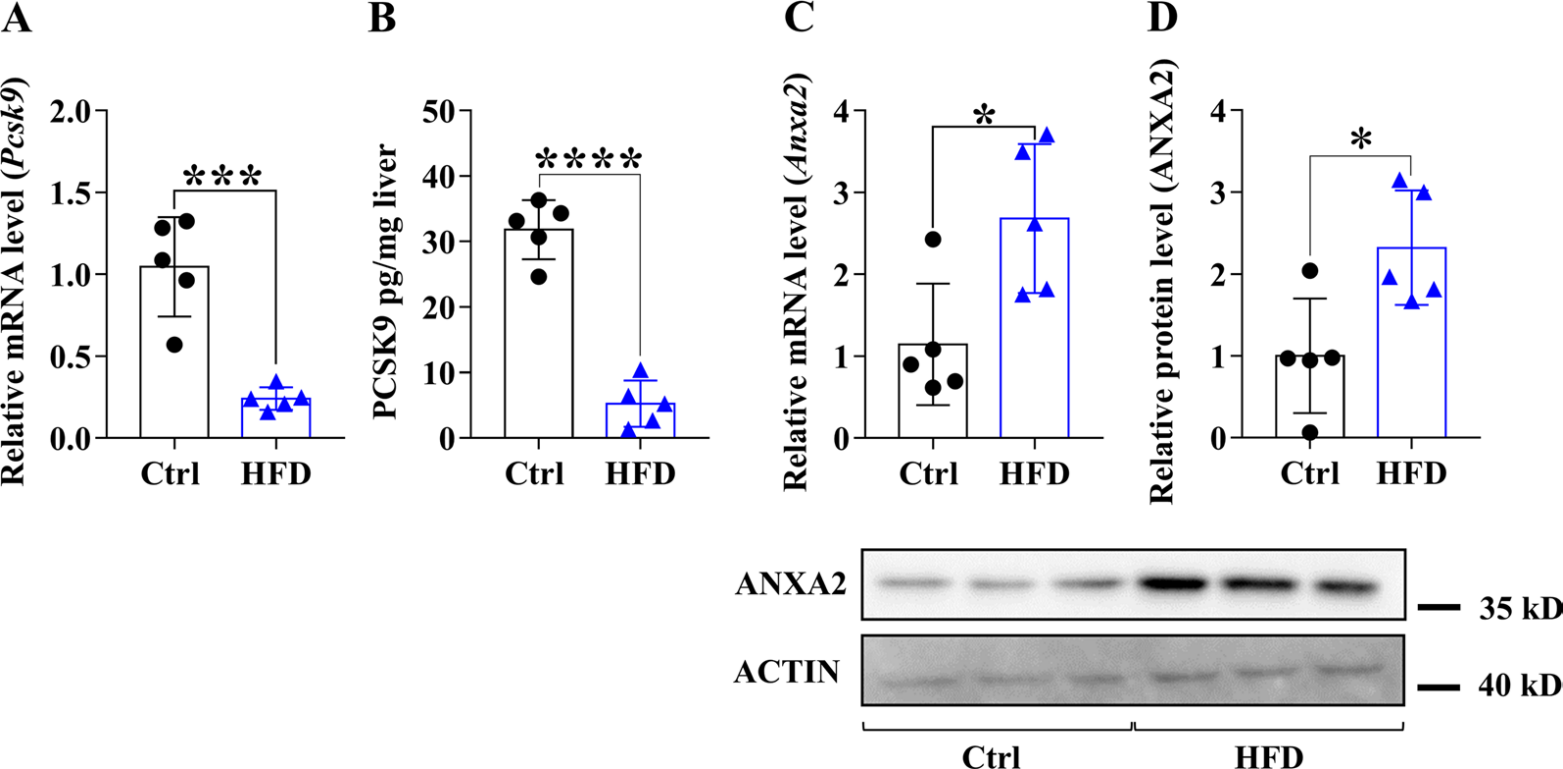
Hepatic mRNA and protein expression of *Pcsk9* and *Anxa2* genes after 20 weeks on HFD. The hepatic expression was assessed at mRNA and protein levels by qPCR/ELISA for PCSK9 (**A, B**) and qPCR/Western blot for ANXA2 (**C, D**). The mRNA level was calculated with 2^−*ΔΔ*Ct^ method. The relative protein level on the blots was determined by ImageJ, using actin as an internal control. Values are reported as mean ± SD; n = 5 in each group. The *p* value was determined by unpaired T-test. *p < 0.05, **p < 0.01, ***p < 0.001

**Fig. 3:**
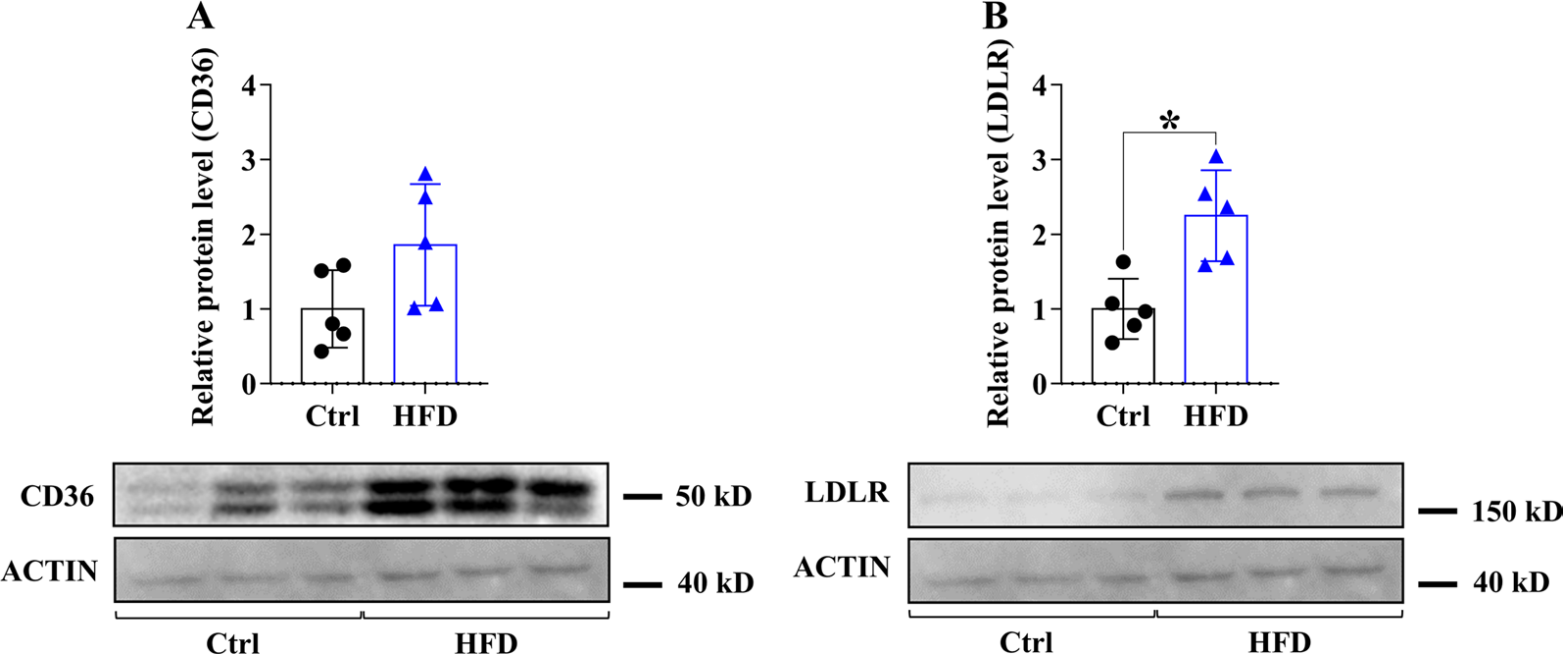
Hepatic protein expression of LDLR and CD36 receptors after 20 weeks on HFD. The hepatic expression of the receptors was measured by Western blot (CD36; **A**, LDLR; **B**). The relative protein level was determined by ImageJ, using actin as an internal control. Values are reported as mean ± SD; n = 5 in each group. The *p* value was determined by multiple T-test (**A** and **B**). *p < 0.05, **p < 0.01, ***p < 0.001

We assessed the hepatic expression of *Pcsk9, Ldlr, Cd36* and *AnxA2* in mice after 20 weeks of HFD feeding, and the results generally show a decrease in PCSK9 while an increase in the others. The expression of *Pcsk9* decreased, while the mRNA level of *AnxA2* increased upon HFD (Fig. 2A, C). PCSK9 protein levels were significantly decreased (Fig. 2B), LDLR and ANXA2 protein levels were significantly elevated (Figs. 2D, 3B) and a non-significant positive change was seen in CD36 protein levels (Fig. 3A) in the HFD group.

### Hepatic lipid profile of WT and PCSK9−/− mice fed with control diet

**Fig. 4:**
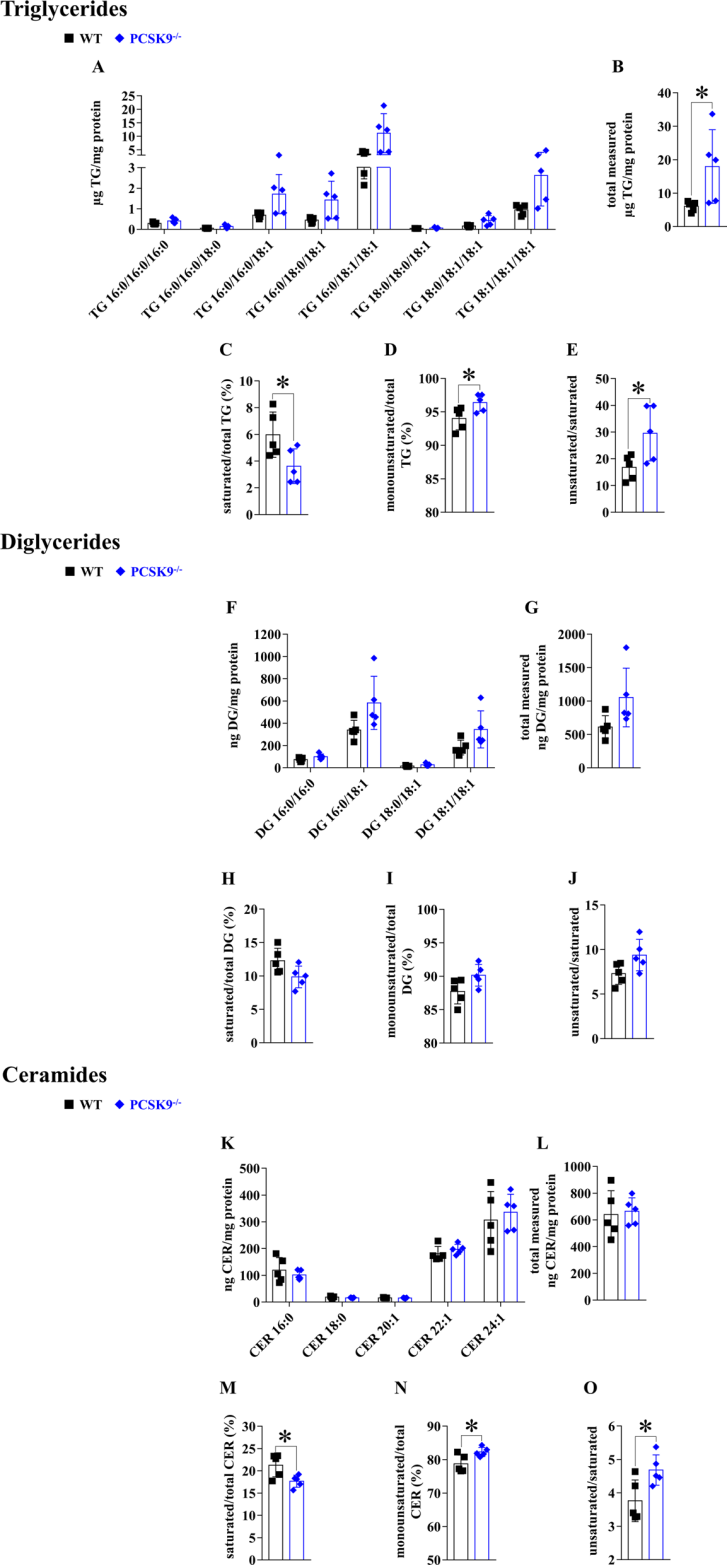
Lipid accumulation in the livers of PCSK9^−/−^ mice on control diet. Lipid composition of the liver was examined by HPLC–MS/MS. The concentration of TG, DG and CER subtypes (**A, F, K**), their total amount (**B, G, L**), the proportion of saturated and monounsaturated TGs, DGs and CERs to total amount (**C, D, H, I, M, N**) and the ratio of monounsaturated and saturated lipids were determined (**E, J, O**). Values are reported as mean ± SD; n = 5 in each group. The *p* value was determined by multiple T-test (**A, F, K**) or unpaired T-test (all others). *p < 0.05

To evaluate the effect of PCSK9 on hepatic lipid accumulation, we compared the relevant lipid contents in the livers of WT and PCSK9^−/−^ mice. Although, the differences in the dominant lipid subtypes (52:2, 54:4, 36:2, 34:1, 22:1, 24:1) were not statistically significant (Fig. 4A, F and K), the total measured TG level was significantly elevated in PCSK9^−/−^ mice (Fig. 4B). The proportion of saturated TGs and CERs decreased (Fig. 4C, M), while the proportion of monounsaturated forms increased (Fig. 4D, N) significantly, and a significant increase was observed in the monounsaturated/saturated ratio of TGs (Fig. 4E) and CERs (Fig. 4O) in the PCSK9^−/−^ mice compared to the WT group. Similar changes were seen when the DG contents were compared (Fig. 4H–J), but without statistical significance.

### Expression of PCSK9, LDLR, CD36 and ANXA2 in the liver of PCSK9−/− mice fed with control diet

**Fig. 5:**
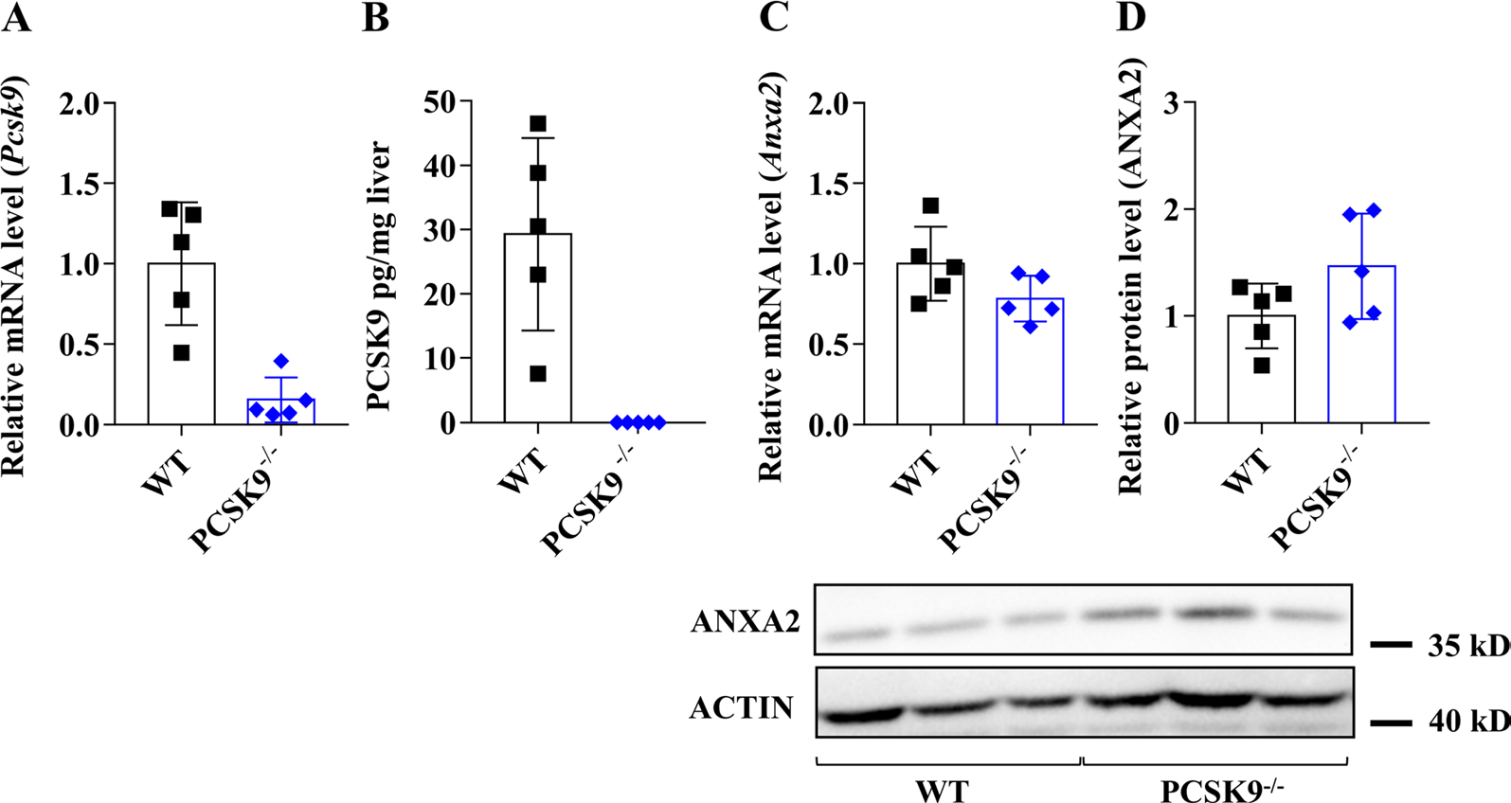
Hepatic gene expression of *Pcsk9* and *Anxa2* genes in PCSK9^−/−^ mice on control diet. The hepatic expression was assessed at mRNA and protein levels by qPCR/ELISA for PCSK9 (**A, B**) and qPCR/Western blot for (ANXA2; **C, D**). The mRNA level was calculated with 2^−*ΔΔ*Ct^ method. The relative protein level on the blots was determined by ImageJ, using actin as an internal control. Values are reported as mean ± SD; n = 5 in each group. The *p* value was determined by multiple T-test (**A** and **B**). *p < 0.05, **p < 0.01, ***p < 0.001

**Fig. 6:**
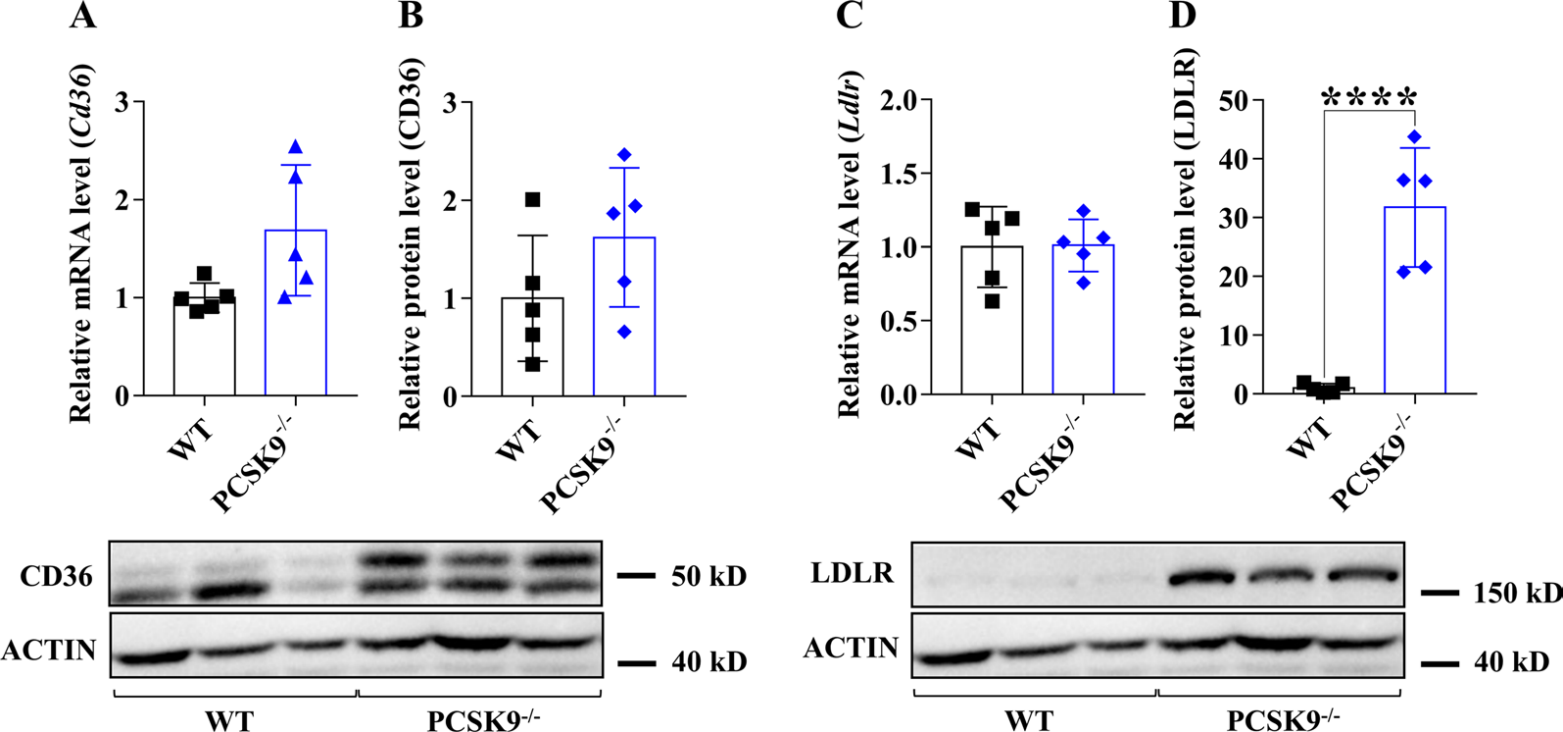
Hepatic gene expression of *Cd36* and *Ldlr* genes in PCSK9^−/−^ mice. Hepatic expression at mRNA level was measured by qPCR and calculated with 2^−*ΔΔ*Ct^ method (**A, C**). The relative protein level on the blots was determined by ImageJ, using actin as an internal control (**B, D**). Values are reported as mean ± SD; n = 5 in each group. The *p* value was determined by multiple T-test (**A** and **B**). *p < 0.05, **p < 0.01, ***p < 0.001

Lack of PCSK9 expression in PCSK9^−/−^ mice was confirmed at both mRNA (Fig. 5A) and protein levels (Fig. 5B). *Ldlr, CD36* and *Anxa2* mRNA levels remained unchanged when compared to the WT group (Figs. 5C, 6A, C). The LDLR protein level was significantly elevated, while the expression of CD36 and ANXA2 proteins was not changed (Figs. 5D and 6B, D).

### Co-localization of PCSK9 with LDLR, CD36 and ANXA2 in HepG2 cells

**Fig. 7:**
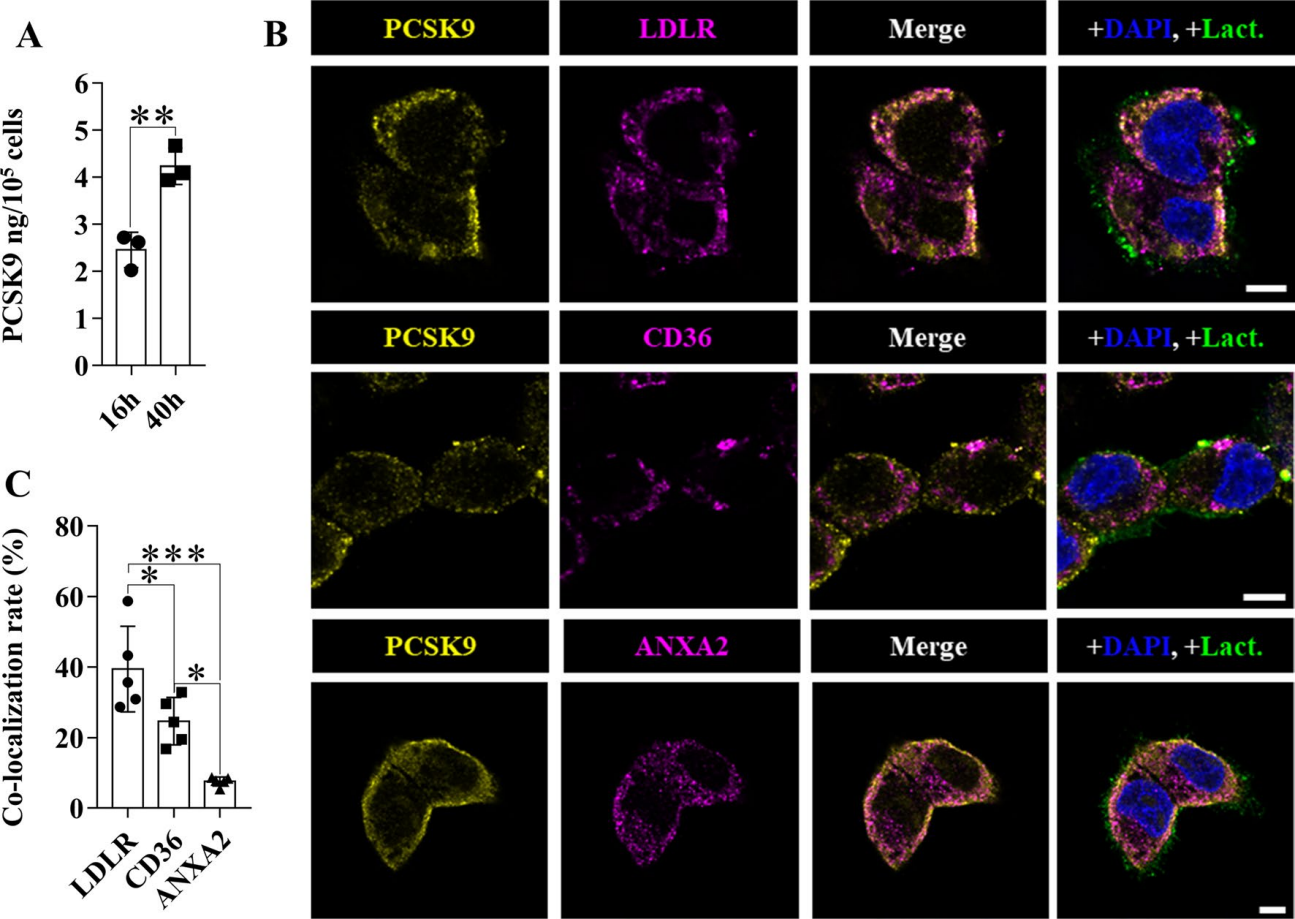
Co-localization of PCSK9 with LDLR, CD36 and ANXA2. HepG2 cells were examined for PCSK9 secretion 16 and 40 h after serum depletion (**A**, n = 3). Cellular localization of PCSK9, LDLR, CD36 and ANXA2 proteins was examined by laser scanning confocal microscopy (**B**). Co-localization rate was used to quantify the degree of co-localization (**C**, n = 5). The scale bar has a width of 5 µm. Values are reported as mean ± SD. The *p* value was determined by unpaired T-test (**A**) or one-way ANOVA combined with Tukey’s multiple comparisons test (**C**). *p < 0.05, **p < 0.01, ***p < 0.001

Co-localization of the PCSK9 protein with the LDLR, CD36 and ANXA2 proteins was tested in HepG2 hepatocellular carcinoma cells using laser scanning confocal microscopy (Fig. 7). The cells showed epithelial-like polygonal morphology in cell culture (Additional file 2: Fig. S4A). The FBS content of the medium was reduced to 2%, which did not cause a significant change in the cell viability at 16 (91.7 ± 3.2%) or 40 (93.7 ± 3.1%) hours (Additional file 2: Fig. S4B). The cell number (16 h: 3.6 × 10^5^ ± 8.8 × 10^4^; 40 h: 4.6 × 10^5^ ± 1.2 × 10^4^) and the confluence (16 h: 55.0 ± 4.4%; 40 h: 67.0 ± 9.0%) were also detected (Additional file 2: Fig. S4B). Since PCSK9 secretion (Fig. 7A) was higher at 40 h after medium change, this time point was chosen to perform the co-localization analysis (Fig. 7B and C). The degree of overlap between the two channels was expressed with co-localization rate by analyzing individual cells as region of interest. PCSK9 showed the highest overlap with LDLR (39.4 ± 12.1%), while it was co-localised with CD36 and ANXA2 in lower rate (24.6 ± 6.7% and 7.6 ± 1.3%, respectively) (Fig. 7C).

## Discussion

We assessed the effect of 20 weeks long HFD on the hepatic lipid content in WT mice, and detected changes in different types of TGs, DGs and CERs. The hepatocellular synthesis of these lipids is affected by the fatty acid supply, which in turn depends on the receptor mediated uptake of free fatty acids (FFAs) and lipoproteins. In this study, we performed experiments to test the role of alterations in LDLR, CD36, PCSK9 and ANXA2 in the changes actually manifested in the hepatic lipid profile after chronic obesogenic HFD and in PCSK9^−/−^ mice. FFA uptake is largely enhanced by the activity of CD36 in the liver [33] and, to some extent, by free fatty acid receptors (FFARs) [34], while the endocytosis of LDL is mediated predominantly by LDLR.

**Fig. 8:**
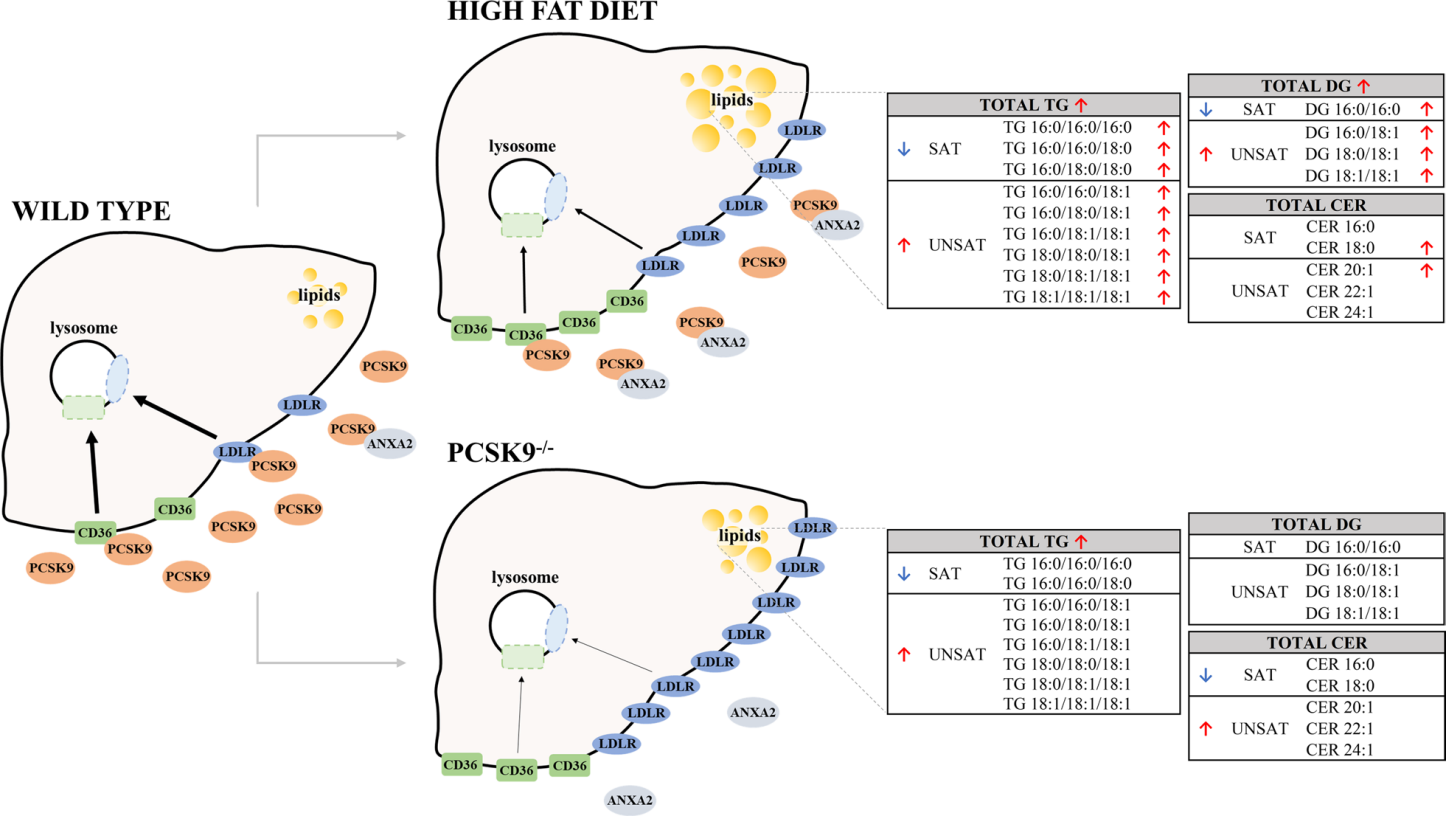
Overview figure summarizing the content of the manuscript. The figure represents changes in PCSK9, LDLR, ANXA2 and CD36 levels after 20 weeks HFD and in PCSK9 gene knockout mice. The quantity of the colour coded proteins is proportional with the amount found under experimental conditions. Changes in different lipid levels are listed in the tables

Our results are summarized on Fig. 8. We show here that in mice, LDLR level increased upon HFD, and accordingly, the expression of PCSK9, a major negative regulator of these receptors [12, 16] decreased while the expression of PCSK9 inhibitor, ANXA2 increased both at protein and mRNA levels. The turnover of the two receptors is affected through direct interaction with PCSK9 and ANXA2, and the co-localization of the four proteins was confirmed in HepG2 cells. Moreover, we demonstrate that WT and PCSK9^−/−^ mice fed with control diets have different lipid profiles, and the changes in PCSK9^−/−^ mice show similarities with those of WT mice in HFD. In both cases (HFD/CTRL, PCSK9^−/−^/CTRL), total TG levels were elevated, and the percentage of SATs were decreased and UNSATs increased. We did not observe equivalent changes in different TGs, DGs and CERs. In both HFD animals (where the level of PCSK9 enzyme was low) and in PCSK9^−/−^ mice (where this enzyme is missing), LDLR protein levels were increased. These two gene products share some regulatory pathways, e.g., both of them are under control of SREBP, which might imply their simultaneous regulation [35].

In PCSK9 deficient mice, there were no significant differences in the amount of individual hepatic lipids, while we found elevations in many lipid species in HFD mice. Although recent knowledge is scarce and mixed, it suggests, that type-dependent alterations and quantity changes of lipids have clinico-pathological relevance. As an example, accumulation of fat droplets can be protective against lipotoxicity, through decreasing the concentration of acyl-CoA in the cytoplasm [36]. On the other hand, fat deposition is a key marker of NAFLD, alongside liver damage and inflammation [37]. This is in line with the low toxicity of oleic acid, which promotes TG accumulation, compared to palmitic acid, which is poorly incorporated into TGs and causes apoptosis [30, 38]. Clinical studies on lipid levels are more consequent, such as one showing that fatty liver diseases can be characterized with different TG amounts and compositions [39]. They revealed elevation of the same TGs (48:0, 50:0, 50:1, 50:2, 54:2, 54:3) in liver diseases as in HFD mice in our experiments (Fig. 1). DG accumulation has a significant role in the development of hepatic insulin resistance [40, 41] and in the progress of NAFLD [42]. In our experiments, total DG amount and specifically 32:0, 34:1, 36:1, 36:2 DGs were increased in the liver of mice fed with HFD (Fig. 1). In agreement with these results, another study found that a single in vitro treatment of hepatoma cells with palmitate increases the level of DG 32:0 [1,2 dipalmitoyl-glycerol (16:0/16:0)] and DG 34:1 [1-palmitoyl-2-oleoyl-glycerol (16:0/18:1)], 45-fold and threefold, respectively [30].

CERs also contribute to the pathogenesis of liver diseases. Elevated levels of CER intermediates (dihydrosphingosine and dihydroceramide) were found in mice fed with diet containing 42% saturated fats/1.5% cholesterol [28]. Pro-apoptotic and pro-inflammatory CERs (16:0, 18:0) were increased in a study with LDLR^−/−^ animals [43]. CER dysregulation was observed in human studies in the late stage of NASH (nonalcoholic steatohepatitis) [44]. In our experiments, C:21:1 and C:18 levels where significantly increased after 20 weeks on HFD.

We investigated the effect of HFD on LDLR and CD36 receptors and their regulators PCSK9 and ANXA2. LDLR levels are regulated by many factors, their level correlates with the plasma concentration of various lipids and lipoproteins [45–47], e.g., in some animal species, hepatic LDLR mRNA level depends on the saturated/unsaturated lipid content of diets [48]. In our present study, obesogenic diet increased hepatic LDLR protein level. Similarly, LDLR protein but not mRNA levels were found to increase in PCSK9^−/−^ mice. However, such phenomenon is not unprecedented as Luo and coworkers found decreased LDLR protein levels without change in mRNA amount in PCSK9 overexpressing transgenic mice [49]. Moreover, statin treatments, known to induce PCSK9 enzyme, have been repeatedly reported to result in increased mRNA and unchanged protein levels of LDLR [50, 51]. In our study, LDLR protein levels were increased in both HFD animals (presenting with lower PCSK9 levels) and in PCSK9^−/−^ mice. Despite the known role of PCSK9 in the control of LDLR recycling, further studies might unveil the involvement of SREBP mediated mechanisms, or even the contribution of epigenetic regulation of LDLR [35].

Recent studies show that increased CD36 levels are accompanied with elevated FFA uptake and TG storage, while hepatocyte-specific *Cd36* deletion protects against HFD-induced liver steatosis, thus this protein might have a crucial role in NAFLD pathology [52, 53]. CD36 has also been linked to metabolic diseases such as insulin resistance, obesity and type 2 diabetes mellitus, with an increasing subpopulation of overweight individuals in Western societies [52]. We found no significant change in CD36 expression, but an increasing trend was observed in the protein level after 20 weeks of treatment.

PCSK9 levels can be modified by many factors, among which, diet, obesity and metabolic diseases are the most important [54]. Its level is regulated by LDL, insulin, fibroblast growth factor 21 (FGF21) and some plant flavonoids (e.g., berberine) mainly through sterol regulatory-element binding protein 2 (SREBP2), mammalian target of rapamycin complex 1 (mTORC1), hepatocyte nuclear factor 1 α (HNF1α), forkhead box O-3 (FoxO3) and miR-27a [55]. We demonstrated that HFD decreases the level of PCSK9 mRNA/protein in mice. PCSK9 can be linked to many pathological conditions, such as insulin resistance, liver steatosis or atherosclerosis [56]. Additionally, it was reported that altered PCSK9 levels contribute to lipid accumulation in hepatocytes [29], but this is the first report on the changes of different lipid types in PCSK9^−/−^ mice compared to WT mice. Comparing the lipid profile of PCSK9^−/−^ and WT mice, we observed increased amounts of total TG and monounsaturated lipids and decreased amounts of saturated lipids. Quantity of saturated and unsaturated CERs and their ratio were also altered in PCSK9^−/−^ mice compared to wild type animals. Thus, we assume that selective accumulation of different lipids depends on the activity of PCSK9.

ANXA2 is a natural inhibitor of PCSK9 [57]. Studies with knockout animals showed that in the absence of this protein, plasma PCSK9 levels are increased, LDLR expression is reduced, and thus, LDL-C levels and the risk for atherosclerosis are higher [58]. Human genetic variants of ANXA2 (rs11633032 and rs17191344) causing decreased quantity of this protein show similar changes to those found in ANXA2 KO mice. ANXA2 is also involved in the regulation of lipid content in liver cells; it is one of the most important markers of steatosis [59]. In our study, the level of this protein was increased, and as expected, the level of LDLR was also increased in HFD fed mice.

## Conclusion

In conclusion, our results show that 20 weeks on HFD and the lack of PCSK9 modulates the amount and type of TGs, DGs and CERs in liver cells. Additionally, our experiments highlight the importance of CD36, LDLR and ANXA2 in alterations of hepatic lipid metabolism and provide additional evidence for the complex regulatory mechanisms mediated by the interplay of these proteins. Our findings raise new questions that we would like to address in our future research. It would be important to elucidate which hepatic lipids are mostly affected by PCSK9 in obesity. Obesity and hypercholesterolemia are known risk factors for cardiovascular diseases, and they are often treated with PCSK9 inhibitors. A better understanding of the consequences of PCSK9 inhibition on the hepatic lipid levels could highlight certain on and off target effects of these drugs.

## Supplementary Information

**Additional file 1:** “High fat diet and PCSK9 knockout modulates lipid profile of the liver and changes the expression of lipid homeostasis related genes”. Protein, TG, DG and CER concentrations of liver pieces.

**Additional file 2:** “High fat diet and PCSK9 knockout modulates lipid profile of the liver and changes the expression of lipid homeostasis related genes”. **Table S1.** Details of antibodies used in experiments.** Fig. S1.** Additional samples, not represented on Figure 2, 3, 5, 6. **Fig. S2**. Original blots. **Fig. S3**. Characterization of HepG2 culture.

## Data Availability

The datasets supporting the conclusions of this article are included within the article (and its additional files).

## Abbreviations

ANXA2: Annexin A2
CD36: Fatty acid translocase scavenger receptor
CER: Ceramide
DG: Diglyceride
FFA: Free fatty acid
FFAR: Free fatty acid receptor
FGF21: Fibroblast growth factor 21
FoxO3: Forkhead box O-3
HFD: High fat diet
HNF1α: Hepatocyte nuclear factor 1α
LDL-C: Low density lipoprotein cholesterol
LDLR: Low density lipoprotein receptor
LRP1: Low density lipoprotein receptor-related protein 1
mTORC1: Mammalian target of rapamycin complex 1
NAFLD: Non-alcoholic fatty liver disease
PCSK9: Serine protease proprotein convertase subtilisin/kexin type 9
SREBP2: Sterol regulatory element-binding protein 2
TG: Triglyceride

## References

[1] Csonka C, Baranyai T, Tiszlavicz L (2017). Isolated hypercholesterolemia leads to steatosis in the liver without affecting the pancreas. Lipids Health Dis.

[2] Weber LW, Boll M, Stampfl A (2004). Maintaining cholesterol homeostasis: sterol regulatory element-binding proteins. World J Gastroenterol.

[3] Pirillo A, Casula M, Olmastroni E, Norata GD, Catapano AL (2021). Global epidemiology of dyslipidaemias. Nat Rev Cardiol.

[4] World Health Organization. Mortality and global health estimates. The top 10 causes of death. 2019. Available in: https://www.who.int/news-room/fact-sheets/detail/the-top-10-causes-of-death

[5] Begg MJ, Sturrock ED, van der Westhuyzen DR (2004). Soluble LDL-R are formed by cell surface cleavage in response to phorbol esters. Eur J Biochem.

[6] Mayne J, Ooi TC (2018). Associations between soluble LDLR and lipoproteins in a white cohort and the effect of PCSK9 loss-of-function. J Clin Endocrinol Metab.

[7] Bjune K, Wierød L, Naderi S (2018). Triciribine increases LDLR expression and LDL uptake through stabilization of LDLR mRNA. Sci Rep.

[8] Krone W, Naegele H, Behnke B, Greten H (1988). Opposite effects of insulin and catecholamines on LDL-receptor activity in human mononuclear leukocytes. Diabetes.

[9] Windler EET, Kovanen PT (1980). The estradiol-stimulated lipoprotein receptor of rat liver. A binding site that membrane mediates the uptake of rat lipoproteins containing apoproteins B and E. J Biol Chem.

[10] Streicher R, Kotzka J (1996). SREBP-1 mediates activation of the low density lipoprotein receptor promoter by insulin and insulin-like growth factor-I. J Biol Chem.

[11] Kwon HJ, Lagace TA (2008). Molecular basis for LDL receptor recognition by PCSK9. Proc Natl Acad Sci USA.

[12] Abifadel M, Varret M, Rabès J (2003). Mutations in PCSK9 cause autosomal dominant hypercholesterolemia. Nat Genet.

[13] Cohen J (2005). Low LDL cholesterol in individuals of African descent resulting from frequent nonsense mutations in PCSK9. Nat Genet.

[14] Osteikoetxea X, Silva A (2022). Engineered Cas9 extracellular vesicles as a novel gene editing tool. J Extracell Vesicles.

[15] Zhang P (2017). PCSK9 as a therapeutic target for cardiovascular disease. Exp Ther Med.

[16] Demers A, Samami S, Lauzier B (2015). PCSK9 induces CD36 degradation and affects long-chain fatty acid uptake and triglyceride metabolism in adipocytes and in mouse liver. Arterioscler Thromb Vasc Biol.

[17] Pepino MY, Kuda O, Samovski D, Abumrad NA (2014). Structure-function of CD36 and importance of fatty acid signal transduction in fat metabolism. Annu Rev Nutr.

[18] Greco D, Kotronen A, Westerbacka J (2008). Gene expression in human NAFLD. Am J Physiol Gastrointest Liver Physiol.

[19] Mayer G, Poirier S, Seidah NG (2008). Annexin A2 is a C-terminal PCSK9- binding protein that regulates endogenous low density lipoprotein receptor levels. J Biol Chem.

[20] Zaid A, Roubtsova A, Essalmani R (2008). Proprotein convertase subtilisin/kexin type 9 (PCSK9): Hepatocytespecific low-density lipoprotein receptor degradation and critical role in mouse liver regeneration. Hepatology.

[21] Seidah NG, Poirier S, Denis M (2012). Annexin A2 Is a natural extrahepatic inhibitor of the PCSK9-induced LDL receptor degradation. PLoS ONE.

[22] Cheung O, Sanyal AJ (2008). Abnormalities of lipid metabolism in nonalcoholic fatty liver disease. Semin Liver Dis.

[23] McClain CJ, Barve S, Deaciuc I (2007). Good fat/bad fat. Hepatology.

[24] Park TS, Hu Y, Noh HL (2008). Ceramide in cardiotoxin in lipotoxic cardiomyopathy. Lipid Res.

[25] Summers SA (2006). Ceramides in insulin resistance and lipotoxicity. Prog Lipid Res.

[26] Bharath LP, Ruan T, Li Y (2015). Ceramide-initiated protein phosphatase 2A activation contributes to arterial dysfunction in vivo. Diabetes.

[27] Kratz M, Cullen P, Kannenberg F (2002). Effects of dietary fatty acids on the composition and oxidizability of low-density lipoprotein. Eur J Clin Nutr.

[28] Deevska GM, Rozenova KA, Giltiay NV (2009). Acid sphingomyelinase deficiency prevents diet-induced hepatic triacylglycerol accumulation and hyperglycemia in mice. J Biol Chem.

[29] Ruscica M, Ferri N, Macchi C (2016). Liver fat accumulation is associated with circulating PCSK9. Ann Med.

[30] Sarnyai F, Somogyi A, Gór-Nagy Z (2020). Effect of cis- and trans-monounsaturated fatty acids on palmitate toxicity and on palmitate-induced accumulation of ceramides and diglycerides. Int J Mol Sci.

[31] Hosios AM, Li Z, Lien EC, Heiden MVG (2018). Preparation of lipid-stripped serum for the study of lipid metabolism in cell culture. Bio Protoc.

[32] Vukman KV, Ferencz A (2020). An implanted device enables in vivo monitoring of extracellular vesicle-mediated spread of pro-inflammatory mast cell response in mice. J Extracell Vesicles.

[33] Xu S, Jay A, Brunaldi K, Huang N, Hamilton JA (2013). CD36 enhances fatty acid uptake by increasing the rate of intracellular esterification but not transport across the plasma membrane. Biochemistry.

[34] Secor JD, Fligor SC, Tsikis ST, Yu LJ, Puder M (2021). Free fatty acid receptors as mediators and therapeutic targets in liver disease. Front Physiol.

[35] Lagace TA (2014). PCSK9 and LDLR degradation: regulatory mechanisms in circulation and in cells. Curr Opin Lipidol.

[36] Zámbó V, Simon-Szabó L, Szelényi P (2013). Lipotoxicity in the liver. World J Hepatol.

[37] Donnelly KL, Smith CI, Schwarzenberg SJ (2005). Sources of fatty acids stored in liver and secreted via lipoproteins in patients with nonalcoholic fatty liver disease. J Clin Invest.

[38] Listenberger LL, Han X, Lewis SE (2003). Triglyceride accumulation protects against fatty acid-induced lipotoxicity. Proc Natl Acad Sci USA.

[39] Alamri H, Patterson NH (2019). Mapping the triglyceride distribution in NAFLD human liver by MALDI imaging mass spectrometry reveals molecular differences in micro and macro steatosis. Anal Bioanal Chem.

[40] Samuel VT, Liu ZX, Qu X (2004). Mechanism of hepatic insulin resistance in non-alcoholic fatty liver disease. J Biol Chem.

[41] Mota M, Banini BA, Cazanave SC, Sanyal AJ (2016). Molecular mechanisms of lipotoxicity and glucotoxicity in nonalcoholic fatty liver disease. Metabolism.

[42] Nikolova-Karakashian M (2018). Alcoholic and non-alcoholic fatty liver disease: Focus on ceramide. Adv Biol Regul.

[43] Kasumov T, Li L, Li M (2015). Ceramide as a mediator of nonalcoholic fatty liver disease and associated atherosclerosis. PLoS ONE.

[44] Montefusco DJ, Allegood JC, Spiegel S, Cowart LA (2018). Nonalcoholic fatty liver disease: Insights from sphingolipidomics. Biochem Biophys Res Commun.

[45] Janice M, Teik CO, Lioudmila T (2018). Associations between soluble LDLR and lipoproteins in a white cohort and the effect of PCSK9 loss-of-function. J Clin Endocrinol Metab.

[46] Yuanyuan Q, Flora T, Mee JK (2020). Phosphatidylinositol-(4,5)-bisphosphate regulates plasma cholesterol through LDL (Low-Density Lipoprotein) receptor lysosomal degradation. Arterioscler Thromb Vasc Biol.

[47] Marcelo AN, Miguel ADM, Marcela ASP (2009). Effects of APOE, APOB and LDLR variants on serum lipids and lack of association with xanthelasma in individuals from Southeastern Brazil. Genet Mol Biol.

[48] Vallim T, Salter AM (2010). Regulation of hepatic gene expression by saturated fatty acids. Prostaglandins Leukot Essent Fatty Acids.

[49] Luo Y, Warren L, Xia D, Jensen H, Sand T, Petras S, Qin W, Miller KS, Hawkins J (2009). Function and distribution of circulating human PCSK9 expressed extrahepatically in transgenic mice. J Lipid Res.

[50] Rashid S, Curtis DE, Garuti R, Anderson NN, Bashmakov Y, Ho YK, Hammer RE, Moon YA, Horton JD (2005). Decreased plasma cholesterol and hypersensitivity to statins in mice lacking Pcsk9. Proc Natl Acad Sci USA.

[51] Ness GC, Zhao Z, Lopez D (1996). Inhibitors of cholesterol biosynthesis increase hepatic low-density lipoprotein receptor protein degradation. Arch Biochem Biophys.

[52] Koonen DP, Jacobs RL, Febbraio M (2007). Increased hepatic CD36 expression contributes to dyslipidemia associated with diet-induced obesity. Diabetes.

[53] Wilson CG, Tran JL (2016). Hepatocyte-specific disruption of CD36 attenuates fatty liver and improves insulin sensitivity in HFD-fed mice. Endocrinology.

[54] Cui CJ, Li S, Li JJ (2015). PCSK9 and its modulation. Clin Chim Acta.

[55] Xia XD, Peng ZS, Gu HM (2021). Regulation of PCSK9 expression and function: mechanisms and therapeutic implications. Front Cardiovasc Med.

[56] Cariou B, Langhi C, Le Bras M (2013). Plasma PCSK9 concentrations during an oral fat load and after short term high-fat, high-fat high-protein and high-fructose diets. Nutr Metab.

[57] Seidah NG, Poirier S, Denis M, Parker R, Miao B, Mapelli C, Prat A, Wassef H, Davignon J, Hajjar KA, Mayer G (2012). Annexin A2 is a natural extrahepatic inhibitor of the PCSK9-induced LDL receptor degradation. PLoS ONE.

[58] Amput P, McSweeney C, Palee S (2019). The effects of proprotein convertase subtilisin/kexin type 9 inhibitors on lipid metabolism and cardiovascular function. Biomed Pharmacother.

[59] Rezaei TM, Rezaei TM, Zamanian AM (2019). ANXA2, PRKCE, and OXT are critical differentially genes in Nonalcoholic fatty liver disease. Gastroenterol Hepatol Bed Bench.

