# Supplementary material for: High fat diet and PCSK9 knockout modulates lipid profile of the liver and changes the expression of lipid homeostasis related genes
Source: Nutr Metab (Lond). 2023 Mar 31;20:19. doi: 10.1186/s12986-023-00738-z (PMC10064771; doi:10.1186/s12986-023-00738-z)
Supplement: Supplementary file 2 — Additional file 2: “High fat diet and PCSK9 knockout modulates lipid profile of the liver and changes the expression of lipid homeostasis related genes”. Table S1. Details of antibodies used in experiments. Fig. S1. Additional samples, not represented on Figure 2, 3, 5, 6. Fig. S2. Original blots. Fig. S3. Characterization of HepG2 culture. [file 12986_2023_738_MOESM2_ESM.docx]

**Supplementary informations**

**High fat diet and PCSK9 knockout modulates lipid profile of the liver and changes the expression of lipid homeostasis related genes**

Krisztina Németh^1,2^, Blanka Tóth^3,4^, Farkas Sarnyai^3^, Anna Koncz^1^, Dorina Lenzinger^1^, Éva Kereszturi^3^, Tamás Visnovitz^1,5^, Brachyahu Meir Kestecher^1,6^, Xabier Osteikoetxea^1,6^, Miklós Csala^3^, Edit I Buzás^1,2,6^, Viola Tamási^3*^

^1^Department of Genetics, Cell- and Immunobiology, Semmelweis University, Nagyvárad tér 4, H-1085, Budapest, Hungary

^2^ELKH-SE Translational Extracellular Vesicle Research Group, Nagyvárad tér 4, H-1085, Budapest, Hungary

^3^Department of Molecular Biology, Semmelweis University, Tűzoltó u. 37-47, H-1094, Budapest, Hungary

^4^Department of Inorganic and Analytical Chemistry, Budapest University of Technology and Economics, Műegyetem rkp. 3, H-1111, Budapest, Hungary

^5^Department of Plant Physiology and Molecular Plant Biology, Eötvös Loránd University, Pázmány Péter sétány 1/A, H-1117, Budapest, Hungary

^6^HCEMM-SE Extracellular Vesicle Research Group, Nagyvárad tér 4, H-1085, Budapest, Hungary

Corresponding author:

* Viola Tamási, tamasi.viola@med.semmelweis-univ.hu

**Supplementary Table 1. Details of antibodies used in experiments**

| **Antigen** | **Host** | **Clonality** | **Clone number** | **Conjugate** | **Manufacturer** |
| --- | --- | --- | --- | --- | --- |
| CD36 | mouse | monoclonal | D-2712 | - | ThermoFisher |
| CD36 | mouse | monoclonal | FA6-152 | - | Abcam |
| LDLR | rabbit | monoclonal | SJ0197 | - | ThermoFisher |
| Annexin A2 | rabbit | monoclonal | JA42-30 | - | ThermoFisher |
| PCSK9 | goat | polyclonal | - | - | Sigma |
| PCSK9 | mouse | monoclonal | 2F1 | - | ThermoFisher |
| mouse IgG |  | polyclonal | - | HRP | Abcam |
| rabbit IgG |  | polyclonal | - | HRP | Abcam |
| mouse IgG | goat | polyclonal | - | Atto488 | Sigma |
| rabbit IgG | goat | polyclonal | - | Atto488 | Sigma |
| rabbit IgG | goat | polyclonal | - | AF700 | ThermoFisher |
| mouse IgG | goat | polyclonal | - | eF570 | ThermoFisher |
| goat IgG | rabbit | polyclonal | - | Cy3 | Sigma |
| mouse IgG | goat | polyclonal | - | Cy5 | ThermoFisher |

**Supplementary Fig. 1. Additional samples, not represented on Figure 2, 3, 5, 6.**

**
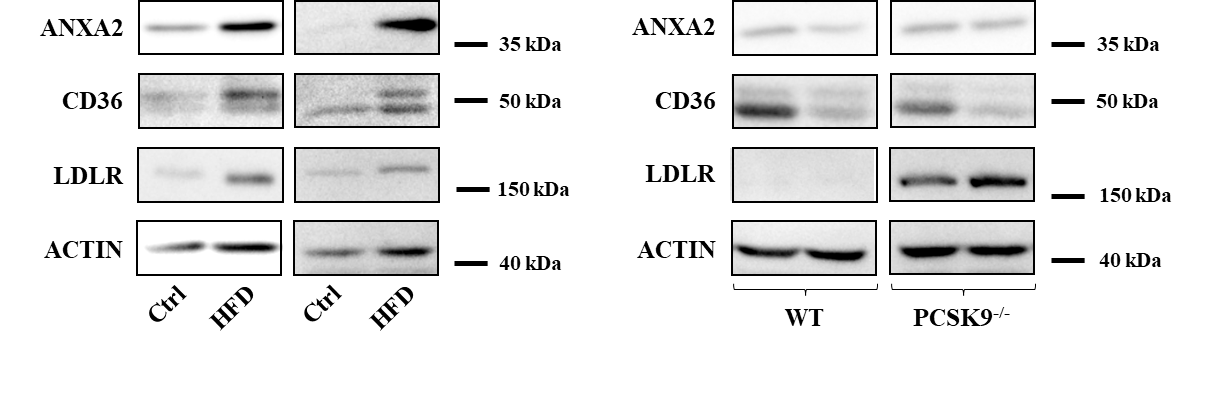
**

**Supplementary Fig. 2. Original blots**


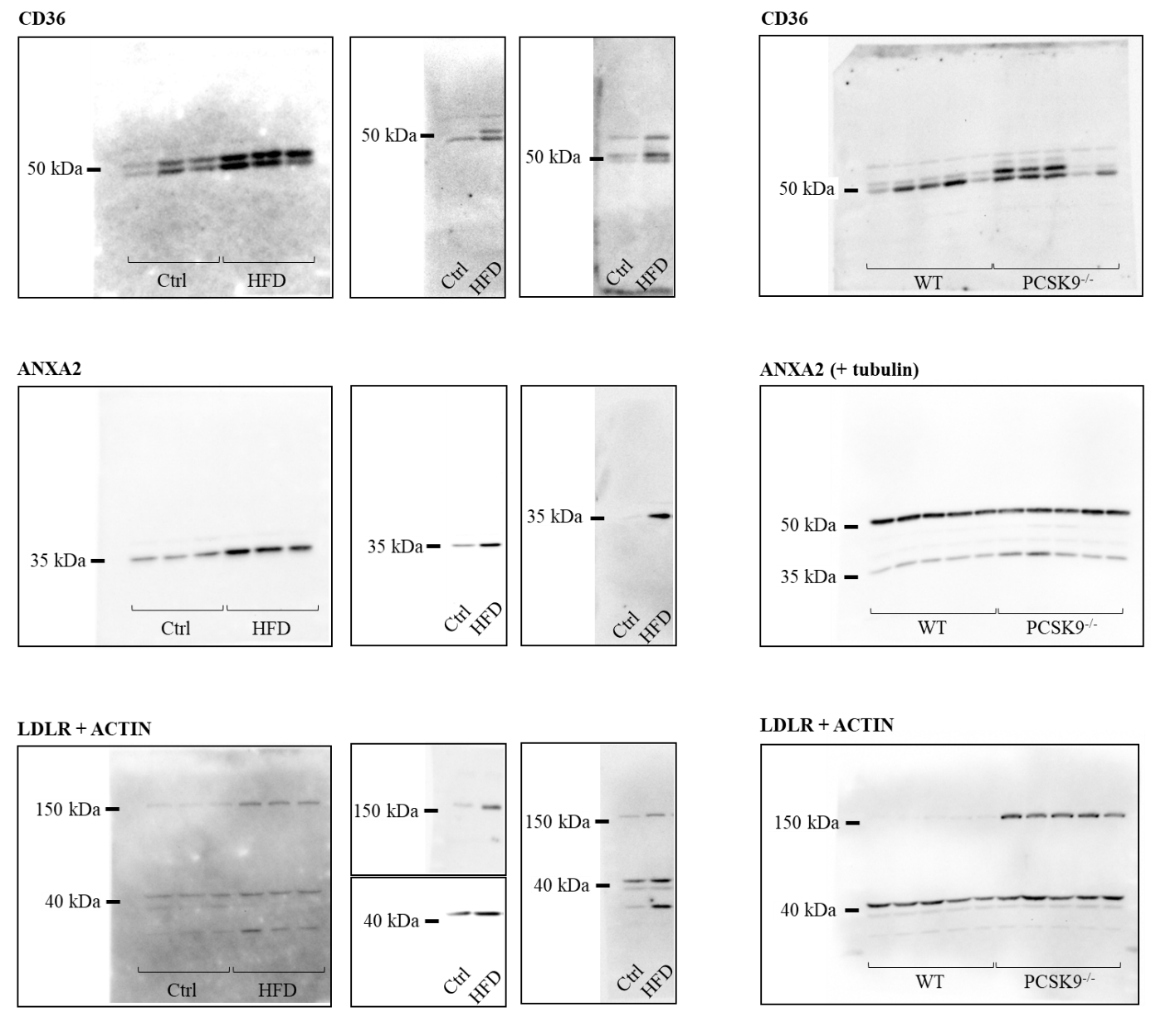


**
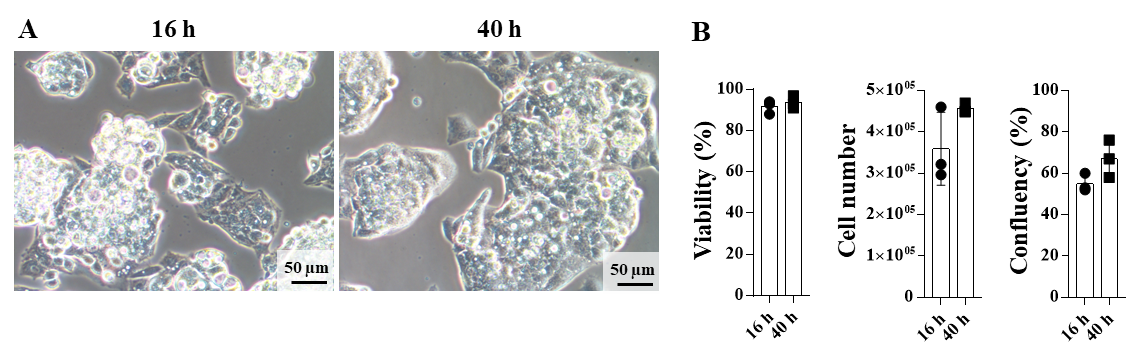
****Supplementary Fig. 3. Characterization of HepG2 culture.** Light microscopic picture of HepG2 cells (A), viability of cultured cells stained with Trypan Blue, cell number and confluency used in the experiments.
